# Intraguild relationships between sympatric predators exposed to lethal control: predator manipulation experiments

**DOI:** 10.1186/1742-9994-10-39

**Published:** 2013-07-10

**Authors:** Benjamin L Allen, Lee R Allen, Richard M Engeman, Luke K-P Leung

**Affiliations:** 1 The University of Queensland, School of Agriculture and Food Sciences, Warrego Highway, Gatton, QLD 4343, Australia; 2 Robert Wicks Pest Animal Research Centre, Biosecurity Queensland, Tor Street, Toowoomba, QLD 4350, Australia; 3 US Department of Agriculture, National Wildlife Research Centre, LaPorte Avenue, Fort Collins, CO 80521-2154, USA

**Keywords:** *Canis lupus dingo*, Dingo, European red fox, *Felis catus*, Feral cat, Mesopredator release, Monitor lizard, Poison baiting, Predator control, Trophic cascade, *Varanus* spp., *Vulpes vulpes*

## Abstract

**Introduction:**

Terrestrial top-predators are expected to regulate and stabilise food webs through their consumptive and non-consumptive effects on sympatric mesopredators and prey. The lethal control of top-predators has therefore been predicted to inhibit top-predator function, generate the release of mesopredators and indirectly harm native fauna through trophic cascade effects. Understanding the outcomes of lethal control on interactions within terrestrial predator guilds is important for zoologists, conservation biologists and wildlife managers. However, few studies have the capacity to test these predictions experimentally, and no such studies have previously been conducted on the eclectic suite of native and exotic, mammalian and reptilian taxa we simultaneously assess. We conducted a series of landscape-scale, multi-year, manipulative experiments at nine sites spanning five ecosystem types across the Australian continental rangelands to investigate the responses of mesopredators (red foxes, feral cats and goannas) to contemporary poison-baiting programs intended to control top-predators (dingoes) for livestock protection.

**Result:**

Short-term behavioural releases of mesopredators were not apparent, and in almost all cases, the three mesopredators we assessed were in similar or greater abundance in unbaited areas relative to baited areas, with mesopredator abundance trends typically either uncorrelated or positively correlated with top-predator abundance trends over time. The exotic mammals and native reptile we assessed responded similarly (poorly) to top-predator population manipulation. This is because poison baits were taken by multiple target and non-target predators and top-predator populations quickly recovered to pre-control levels, thus reducing the overall impact of baiting on top-predators and averting a trophic cascade.

**Conclusions:**

These results are in accord with other predator manipulation experiments conducted worldwide, and suggest that Australian populations of native prey fauna at lower trophic levels are unlikely to be negatively affected by contemporary dingo control practices through the release of mesopredators. We conclude that contemporary lethal control practices used on some top-predator populations do not produce the conditions required to generate positive responses from mesopredators. Functional relationships between sympatric terrestrial predators may not be altered by exposure to spatially and temporally sporadic application of non-selective lethal control.

## Introduction

Terrestrial top-predators can play important roles in structuring food webs and ecosystems through their consumptive (e.g. predation) and non-consumptive (e.g. fear, competition) effects on sympatric mesopredator and herbivore species [1]. Cessation of lethal control and active restoration of top-predators has resulted in biodiversity benefits at lower trophic levels in some systems [2,3]. Perhaps the most widely-known example of positive ecological outcomes arising from the restoration of top-predators is the reintroduction of gray wolves *Canis lupus* to the Greater Yellowstone Ecosystem in North America. Wolf restoration has coincided with remarkable changes to faunal and floral communities there ([4]; but see [5-7]), but has also increased conflict between humans and wolves [8-10].

Human-predator conflicts occur worldwide and are growing in frequency, severity and geographical distribution [11]. Predator attacks on livestock or managed game are a common cause of human-predator conflict [2,11]. Contemporary management of many top-predators now relies on finding the right balance between the conservation of top-predator populations and the alleviation of damage to livestock and game. Lethal control or harvesting of top-predators is one commonly-practiced way of mitigating human-predator conflict [12,13], which may be achieved by hunting (trapping and/or shooting) or poisoning in different parts of the world. In places where top-predator populations are robust and common, their strategic lethal control (or periodic, temporary suppression) might facilitate profitable livestock production while retaining the important functional roles of predators in limiting, suppressing or regulating sympatric species. Such management approaches may not be suitable for top-predators that are uncommon or threatened, which are usually unable to withstand even low levels of human-caused mortality. Given that conflicts between humans and top-predators are likely to continue, a greater understanding of the trophic effects of top-predator control practices on sympatric species is needed to identify appropriate predator control strategies and harvest thresholds in livestock production areas. Although the ecological effects of top-predator extirpation (and recovery) are relatively well understood, the indirect effects of periodic top-predator suppression have received less attention [13-15]. However, small reductions in top-predator populations are predicted to produce disproportionately large positive responses from mesopredators [16,17]. Knowledge of the ecological relationships between humans, top-predators, mesopredators, native prey and livestock is lacking [1,3,18], but can highlight sustainable solutions for coexistence between them. Bears, big cats and wild canids pose particular management challenges because their habitat and food requirements often overlap with humans [13].

Dingoes (*Canis lupus dingo* and hybrids) are the largest terrestrial predator on mainland Australia (typically 12–20 kg) and are the most closely related wild canid to gray wolves [19]. Dingoes were introduced to Australia by humans via south-east Asia about 5,000 years ago, but they are nevertheless considered by many people to be native, or at the very least, an integral component of contemporary Australian ecosystems. Dingoes were ubiquitous across the continent by the time European colonisation of Australia began in the late 1700 s [20]. Dingoes were once effectively exterminated from <25% of Australia by the early-mid 1900s to enable viable sheep *Ovis aries* and goat *Capra hircus* production. However, dingoes are now present (albeit in various densities) across almost all mainland biomes, they are common or increasing in most areas, and their populations are often controlled through periodic lethal control programs for the protection of livestock and some threatened fauna [21]. There are probably more dingoes now than at any other time in Australia’s ecological history in spite of their lethal control [22]. This is because dingoes are beneficiaries of the increased availability of artificial water sources associated with the historical expansion of rangeland pastoralism and the introduction of several other exotic prey species, notably European rabbits *Oryctolagus cuniculus*[20]. Despite their burgeoning population, the genetic identity of dingoes is changing through hybridisation with domestic dogs brought to Australia since European settlement. Thus, some specific genotypes of the Australian dingo population are in decline [23]. Genetic identity issues aside, faunal biodiversity conservation is expected by some to be compromised by lethal dingo control through its perceived indirect positive effects on lower-order predators, or mesopredators (e.g. [16,24]). A few small-scale observational studies have reported positive responses of mesopredators to lethal dingo control (e.g. [25-27]). Other snap-shot, observational or correlative studies have sometimes reported negative relationships between dingoes and sympatric mesopredators or positive relationships between dingoes and some threatened fauna (reviewed in [28,29]). These have fuelled much debate and speculation that contemporary lethal dingo control practices might indirectly enhance mesopredator populations and ultimately harm threatened fauna.

Unfortunately, almost all of the relevant studies underpinning such speculation suffer from methodological design and application issues which render the data either invalid, unreliable or at best inconclusive [30]. These inescapable issues continue to be ignored (e.g. [31]), and despite the weak and inconclusive state of the literature, cessation of lethal dingo control has been recommended by some as the preferred management action to suppress mesopredators through trophic effects (e.g. [16,32,33]). Populations of invasive red foxes (*Vulpes vulpes*, up to 8 kg), invasive feral cats (*Felis catus*, up to 7 kg) and native goannas (or monitor lizards, *Varanus* spp., up to 5 kg) are predicted to respond most positively to dingo control [16,34]. These mesopredators can often have detrimental effects on native and threatened fauna. Because observational and correlative data have no power whatsoever to demonstrate causal processes [35,36], results from manipulative experiments (which *can* provide conclusive data) are sorely needed to substantiate these speculations and provide a defensible evidence-base for predator management [13,28,30,37]. Considering the applied nature of the issue, manipulative experiments which evaluate the overall outcomes of contemporary top-predator control practices (or cessation of control) on mesopredators should be valuable to managers of predators and the native prey fauna they each threaten.

**Figure 1:**
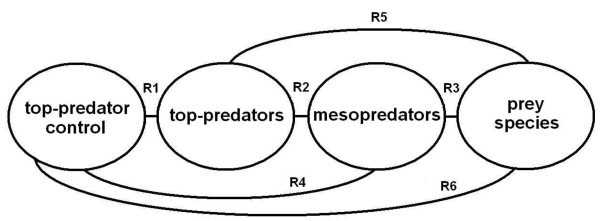
**Schematic representation of the six primary relationships of interest (R1–R6) between top-predator control and prey species at lower trophic levels (see **[15]**).**

We therefore used a series of predator manipulation experiments – those with the highest level of inference logistically achievable in open rangeland areas [28,30] – to determine (1) whether or not sympatric mesopredator abundances were higher, or became higher in areas subjected to top-predator control, (2) whether or not sympatric mesopredator activity levels increased immediately after top-predator control, and (3) how sympatric mesopredator abundance trends correlated with top-predator abundance trends over time. There are six primary relationships between top-predator control and prey fauna (Figure 1). Conceptually, our primary aim was not to investigate the relationship between dingoes and mesopredators (R2 in Figure 1). Rather, we experimentally assessed whether or not fox, cat or goanna populations exhibited an overall benefit from contemporary poison-baiting programs aimed at controlling dingoes (R4 in Figure 1). Comparisons were made between a series of paired baited and unbaited areas monitored over time (see Methods for details of study sites and design, predator population monitoring techniques and analytical approaches). These experiments were conducted across the breadth of the beef-cattle rangelands of Australia. We report the results of the only studies to date with the capacity to demonstrate the effects of lethal dingo control on sympatric mesopredators [30], collectively comprising one of the largest geographic scale predator manipulation experiments conducted on any species anywhere in the world [18].

## Results

### Overall patterns in abundance

**Figure 2:**
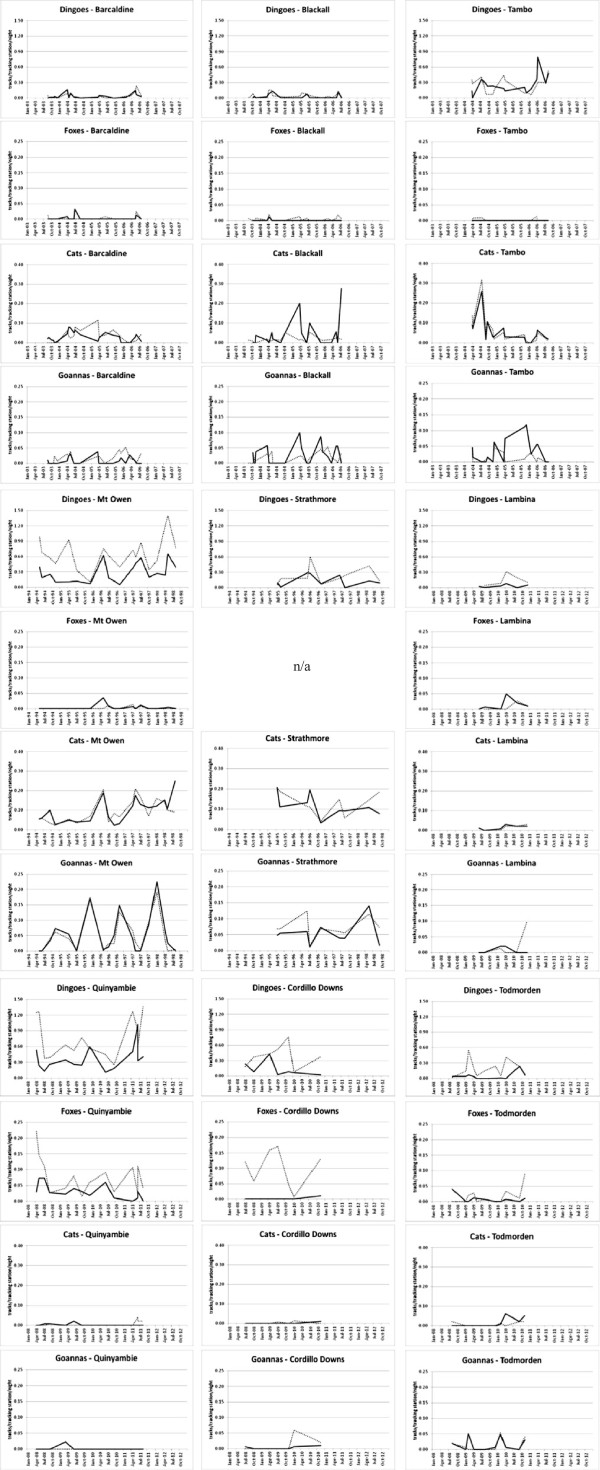
Abundance trends of dingoes, foxes, cats and goannas in paired baited (solid lines) and unbaited (dotted lines) treatment areas at nine sites across Australia.

We found no indication that mean dingo, fox or cat passive tracking index (PTI) values were substantially greater or became greater in areas subjected to periodic poison-baiting for dingoes (Figure 2). The overall mean PTI values for dingoes were demonstrably less (43–77% lower) in baited areas than in paired unbaited areas for five of the six experimental sites and one of the three Blackall sites (Table 1). PTI values for dingoes were similar in both treatments at all other sites. Overall mean fox PTI values were also demonstrably less in the baited areas for four of the eight sites where foxes are found, and were similar in both treatments at the remaining four sites. At no site was a demonstrable difference found between the baited and unbaited areas in overall mean PTI values for cats (Table 1). Treatment differences in PTI values for goannas were found at three sites, with higher overall mean PTI values in the baited area at two of these sites (Table 1). Thus, the greater overall abundance of goannas in the baited areas of Tambo and Blackall were the only two instances (of 26 possible site × mesopredator combinations) where a sympatric mesopredator was detected more frequently in a paired dingo-baited area at any site. Stratifying the data by season indicated that each predator was in similar abundance in both baited and unbaited areas in 76 of 88 cases (Table 2). In every case where demonstrable differences between treatments were detected for any season (12 of 88 possible site × mesopredator × season combinations), dingoes, foxes, cats and goannas were each in greater abundance in unbaited areas (Table 2), indicating no overall benefit to mesopredators from top-predator control.

**Table 1:** **Average PTI values and *p *values for dingoes and sympatric predators at nine sites across Australia (PTI values are from all surveys in paired dingo-baited and unbaited areas; see Table **2**for seasonal breakdown)**

**Species**	**Site**	**PTI values**		***P***
		**Baited**	**Unbaited**	
Dingo	Barcaldine	0.0378	0.0422	0.644
	Blackall	0.0271	0.0474	0.016
	Cordillo	0.1386	0.3871	0.053
	Lambina	0.0340	0.1480	0.046
	Mt Owen	0.2858	0.6732	<0.001
	Quinyambie	0.3686	0.7579	0.002
	Strathmore	0.1333	0.2289	0.119
	Tambo	0.2613	0.2406	0.688
	Todmorden	0.0455	0.1818	0.016
	ALL SITES	0.1492	0.3018	0.001
Fox	Barcaldine	0.0022	0.0022	1.000
	Blackall	0.0005	0.0052	0.004
	Cordillo	0.0014	0.1000	0.005
	Lambina	0.1800	0.0080	0.394
	Mt Owen	0.0042	0.0016	0.235
	Quinyambie	0.0300	0.0786	0.005
	Strathmore	N/A	N/A	N/A
	Tambo	0.0000	0.0019	0.083
	Todmorden	0.0082	0.0173	0.325
	ALL SITES	0.0080	0.0265	0.190
Cat	Barcaldine	0.0300	0.0291	0.901
	Blackall	0.0410	0.0148	0.110
	Cordillo	0.0014	0.0029	0.604
	Lambina	0.0160	0.0140	0.621
	Mt Owen	0.0979	0.0963	0.888
	Quinyambie	0.0029	0.0064	0.373
	Strathmore	0.1167	0.1322	0.461
	Tambo	0.0563	0.0631	0.312
	Todmorden	0.0127	0.0064	0.341
	ALL SITES	0.0429	0.0397	0.584
Goanna	Barcaldine	0.0100	0.0200	0.030
	Blackall	0.0324	0.0071	0.017
	Cordillo	0.0040	0.0160	0.284
	Lambina	0.0133	0.0367	0.606
	Mt Owen	0.0700	0.0629	0.260
	Quinyambie	0.0025	0.0000	0.351
	Strathmore	0.0767	0.0867	0.678
	Tambo	0.0483	0.0180	0.020
	Todmorden	0.0167	0.0122	0.483
	ALL SITES	0.0321	0.0255	0.336

**Table 2:** Average PTI values and *p *values obtained from t-tests for differences in the relative abundance of sympatric predators (assessed separately for each season) between baited and unbaited areas at nine sites in Australia (^greater abundance in unbaited areas; *greater abundance in baited areas; #equal abundance in both baited and unbaited areas; X = insufficient data to calculate *p*)

	**Autumn (March-May)**			
**Site**	**Dingo**	**Fox**	**Cat**	**Goanna**
Barcaldine	0.8200	0.6800^	0.8000*	0.6300^
Blackall	0.2000	0.0509	0.2600	0.1100*
Cordillo	X#	X^	X#	X#
Lambina	X^	X*	X*	X*
Mt Owen	0.0064^	0.2500*	0.2000^	0.6700*
Quinyambie	0.0205^	0.0663^	X#	0.3900*
Strathmore	X^	X#	X^	X*
Tambo	0.6400^	0.1700^	0.9000*	0.0980*
Todmorden	0.2000^	0.4200^	0.4200*	X#
	**Winter (June-August)**			
	**Dingo**	**Fox**	**Cat**	**Goanna**
Barcaldine	0.3900^	0.7900*	0.5800^	N/A
Blackall	0.2200^	0.1800^	0.3200*	
Cordillo	0.5600^	0.1100^	0.5000^	
Lambina	0.3700^	1.0000#	X#	
Mt Owen	0.0019^	X#	0.4600*	
Quinyambie	0.0775^	0.0077^	0.4000^	
Strathmore	0.2800^	X#	0.9500*	
Tambo	0.7400*	0.3900^	0.3800^	
Todmorden	0.5800^	0.5000*	0.5000^	
	**Spring (September-November)**			
	**Dingo**	**Fox**	**Cat**	**Goanna**
Barcaldine	0.4200*	X#	0.5400*	0.1000^
Blackall	0.1500^	0.3600^	0.6800*	0.1900*
Cordillo	0.0665^	0.0726^	0.4200*	0.4200^
Lambina				
Mt Owen	0.0062^	X#	0.9300^	0.5200*
Quinyambie	0.2300^	1.0000#	X#	X#
Strathmore	0.6600^	X#	0.4400^	0.5000^
Tambo	0.0916*	X#	0.1800^	0.1500^
Todmorden	0.8000^	0.4200^	0.5000*	0.5000^
	**Summer (December-February)**			
	**Dingo**	**Fox**	**Cat**	**Goanna**
Barcaldine	0.5300^	X#	0.5300^	0.2200^
Blackall	0.5000^	0.5000^	0.0001^	0.3000*
Cordillo	X^	X^	X^	X^
Lambina	0.0577^	X#	1.0000#	0.5600^
Mt Owen	X^	X#	X^	X*
Quinyambie	X*	X^	X#	X#
Strathmore				
Tambo	0.1400*	X#	0.5000*	1.0000#
Todmorden	0.1200^	0.3900^	X#	0.4100*

### Short-term behavioural responses

**Figure 3:**
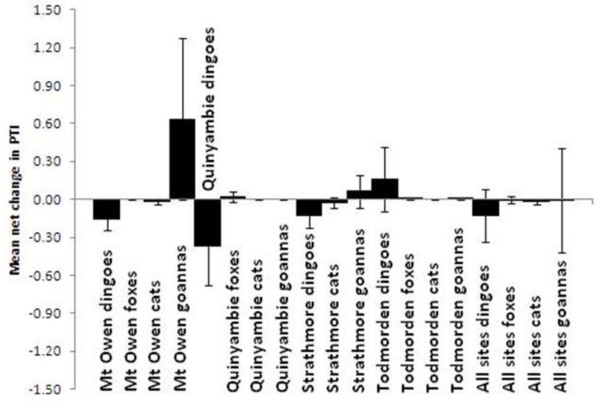
Mean net changes in predator PTI (and 95% confidence intervals) between pre- and post-baiting surveys (conducted within four months of baiting) at Mt Owen (N = 8), Quinyambie (N = 4), Strathmore (N = 5), Todmorden (N = 5) and all sites combined (N = 25), showing no evidence of rapid increases in fox, cat or goanna PTI following dingo control.

A total of 25 baiting events from all sites included post-baiting surveys conducted within four months of baiting from both treatments (mean number of days since baiting = 51). Assessing the short-term responses of predators between surveys conducted just prior and subsequent to baiting showed no indication of short-term behavioural increases or decreases in mesopredator PTI following these dingo control events (Tables 3 and 4). There was a demonstrable effect of time on dingoes (F_1,18_ = 14.37, *p* = 0.0013) and cats (F_1,18_ = 3.91, *p* = 0.0636), and a demonstrable effect of treatment on dingoes only (F_1,18_ = 46.01, *p* = 0.0001). However, no demonstrable time x treatment interactions were detected for any species (Table 3), indicating that the observed post-baiting decreases in dingo and cat PTIs were independent of treatment. No effects of time, treatment or their interaction were found for foxes or goannas (Tables 3 and 4). We likewise found no indication of short-term behavioural increases of mesopredators by assessing the mean net changes in predator PTI between pre- and post-baiting surveys at Mt Owen (N = 8), Quinyambie (N = 4), Strathmore (N = 5) or Todmorden (N = 5) (Figure 3). An insufficient number of pre- and post-baiting pairs to reliably run this analysis were obtained from the other sites. Using this approach, demonstrable changes were only found for dingoes, which were reduced by baiting, and only at Mt Owen, Quinyambie and Strathmore, but not Todmorden. Combining the data from all sites showed no overall short-term changes in PTI for any predator except cats, which slightly declined following dingo control (Figure 3).

**Table 3:** Effects of time (T; pre- or post-baiting), treatment (B; baited or unbaited), and time x treatment interactions on the short-term responses of dingoes, foxes, cats and goannas to lethal dingo control events (E) where post-baiting surveys were conducted within four months of dingo-control (mean = 51 days)

		**Dingo**		**Fox**		**Cat**		**Goanna**	
**Source**	**df**	**F**	***p***	**F**	***p***	**F**	***p***	**F**	***p***
E	18 (14)								
T	1	14.37	0.0013	0.39	0.5424	3.91	0.0636	0.07	0.7893
T × E*	18 (14)								
B	1	46.01	0.0001	2.22	0.1580	0.92	0.3504	0.52	0.4818
B × E*	18 (14)								
T × B	1	0.12	0.7315	0.33	0.5755	0.31	0.5829	2.41	0.1381
T × B × E*	18 (14)								

* = Error terms (with adjusted values for those of foxes in parentheses). See Table 4 for mean PTI values and sample sizes used in these analyses.

**Table 4:** Sample sizes and mean PTI values used in the 2-factor (time, T; treatment, B) repeated measures ANOVA assessing the short-term responses of dingoes, foxes, cats and goannas to lethal dingo-control programs (sample sizes for foxes in parentheses)

			**N=**	**Dingo**	**Fox**	**Cat**	**Goanna**
T	Pre-baiting		38 (30)	0.408	0.025	0.064	0.070
	Post-baiting		38 (30)	0.238	0.022	0.046	0.055
B	Baited		38 (30)	0.189	0.014	0.052	0.027
	Unbaited		38 (30)	0.456	0.032	0.058	0.030
	**Time**	**Treatment**	**N=**	**Dingo**	**Fox**	**Cat**	**Goanna**
T × B	Pre-baiting	Baited	19 (15)	0.267	0.017	0.060	0.028
		Unbaited	19 (15)	0.549	0.032	0.069	0.026
	Post-baiting	Baited	19 (15)	0.112	0.011	0.045	0.026
		Unbaited	19 (15)	0.364	0.032	0.048	0.034

### Longer-term abundance trends

We found mixed, but typically neutral results for longer-term correlations in PTI trends between predators (Table 5, Figure 2). Foxes in the unbaited areas at Barcaldine and Blackall and in the baited area of Mt Owen were positively correlated with dingoes, whereas they were negatively correlated in the baited area at Quinyambie only (where overall fox abundance in the unbaited area was more than double that of the baited area; Table 1). The only dingo-cat correlation different from zero was a positive relationship observed in the baited area at Mt Owen, which also contributed the only two dingo-goanna correlations distinguishable from zero, which were both negative (Table 5). Overwhelmingly however, temporal correlations of dingo PTI values with those of sympatric mesopredators were indistinguishable from zero in the vast majority of cases (Table 5, Figure 2).

**Table 5:** **Correlations (*r*) and *p *values of fox, cat and goanna PTI values with those for dingoes in baited and unbaited areas**

	**Fox r (*p*)**		**Cat r (*p*)**		**Goanna r (*p*)**	
**Site**	**Baited**	**Unbaited**	**Baited**	**Unbaited**	**Baited**	**Unbaited**
Barcaldine	0.164 (0.455)	0.719 (<0.001)	0.234 (0.265)	−0.159 (0.486)	0.157 (0.547)	−0.170 (0.515)
Blackall	0.264 (0.247)	0.390 (0.066)	−0.217 (0.344)	−0.008 (0.971)	−0.311 (0.224)	0.299 (0.243)
Cordillo	−0.355 (0.435)	0.222 (0.632)	−0.355 (0.435)	−0.273 (0.553)	−0.527 (0.362)	−0.796 (0.107)
Lambina	0.624 (0.281)	0.144 (0.818)	0.670 (0.216)	0.473 (0.421)	0.115 (0.927)	−0.543 (0.634)
Mt Owen	0.613 (0.005)	0.090 (0.713)	0.689 (0.001)	0.273 (0.259)	−0.539 (0.047)	−0.670 (0.009)
Quinyambie	−0.558 (0.038)	0.412 (0.143)	−0.299 (0.299)	0.172 (0.558)	−0.032 (0.939)	X
Strathmore	N/A	N/A	0.373 (0.323)	−0.108 (0.782)	0.724 (0.485)	0.984 (0.114)
Tambo	X	0.245 (0.360)	0.121 (0.656)	0.240 (0.370)	0.087 (0.788)	−0.065 (0.840)
Todmorden	−0.197 (0.561)	0.080 (0.814)	0.101 (0.787)	−0.416 (0.203)	−0.136 (0.723)	−0.471 (0.200)

X = insufficient data.

### Triangular relationships

**Figure 4:**
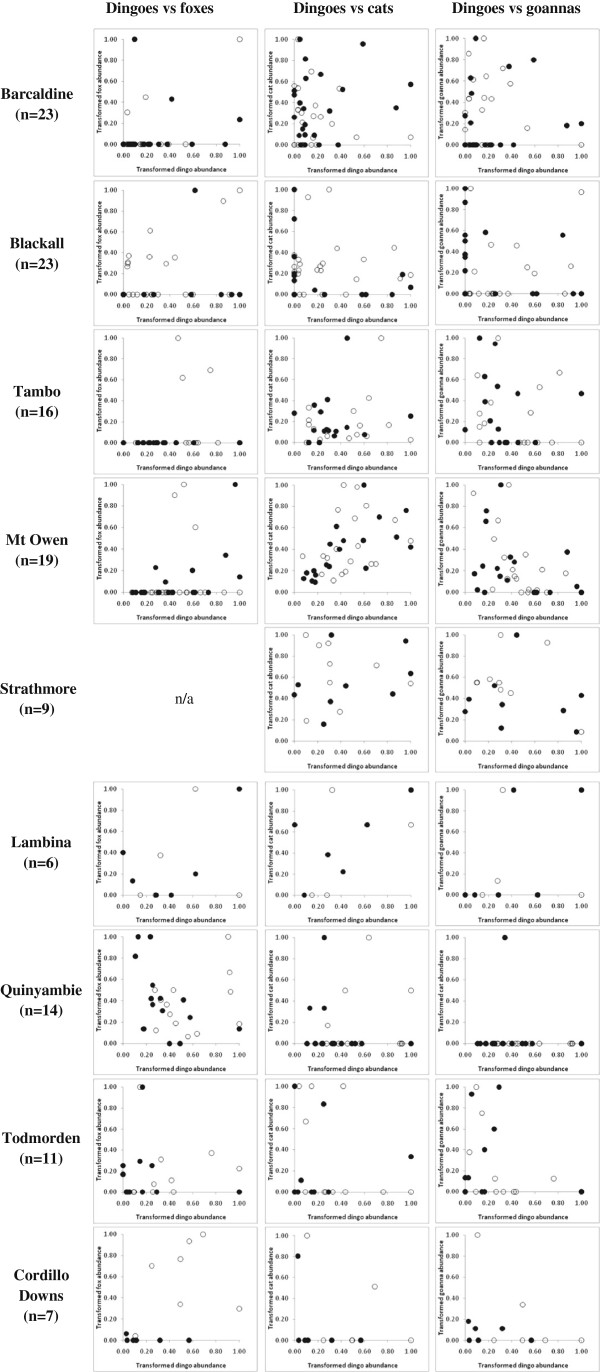
**Correlative relationships between dingo and fox, cat and goanna PTI in baited (solid marks) and unbaited (hollow marks) treatment areas at nine sites across Australia, as generated using the approach described by Johnson and VanDerWal **[38]**, showing the typical absence of triangular relationships amongst sympatric predators.**

**Figure 5:**
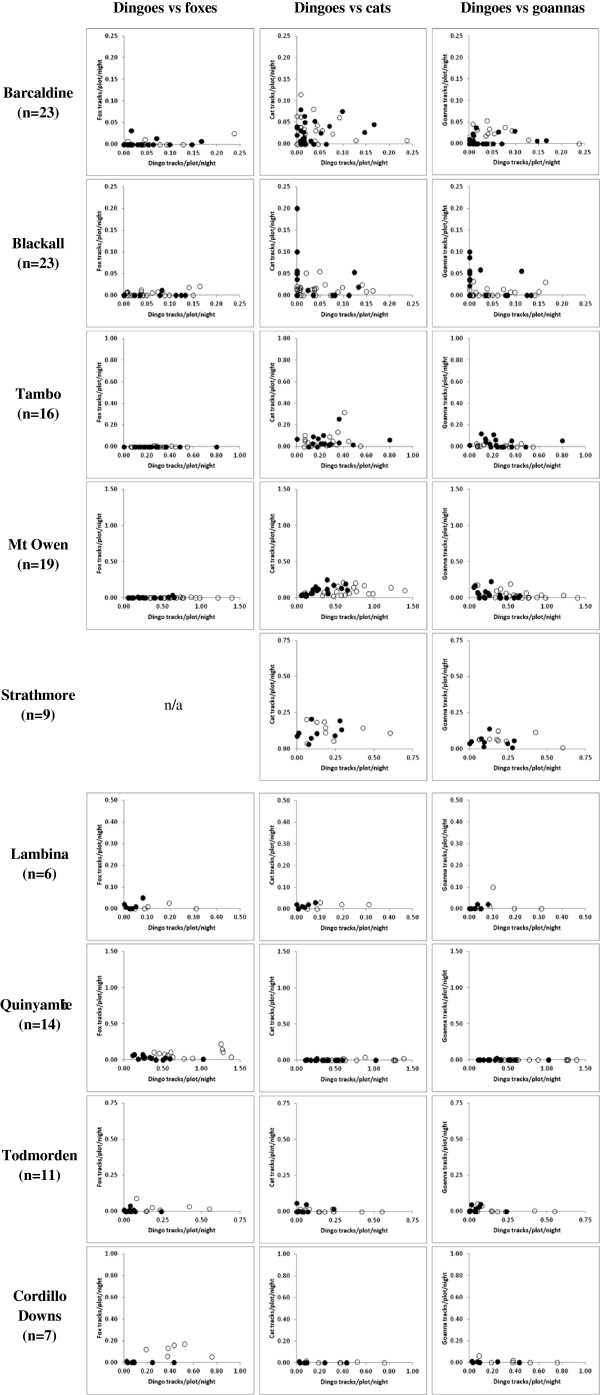
**Correlative relationships between dingo and fox, cat and goanna PTI in baited (solid marks) and unbaited (hollow marks) treatment areas at nine sites across Australia, as generated using the approach described by Johnson and VanDerWal **[38]**, but without data transformation, showing the typical absence of triangular relationships amongst sympatric predators.**

Analysing our data using an alternative approach described by Johnson and VanDerWal [38] provided inconclusive evidence of negative relationships between dingoes and any of the mesopredators we assessed at our study sites (Figure 4). The shape of the relationships also changed depending on whether or not the data were transformed (compare Figures 4 and 5). However, both approaches were consistent in showing that ‘triangular relationships’ (*sensu*[38]) typically did not exist between dingoes and foxes, cats or goannas at the sites we monitored. In other words, we found little evidence that variability in fox, cat or goanna PTI decreased as dingo PTI increased using this approach. In contrast, mesopredator PTI typically varied little despite relatively large fluctuations in dingo PTI (Figures 4 and 5), suggesting that mesopredator abundances fluctuate independent of dingo abundances or dingo control (as shown in Figure 2). Regardless, we question the utility and reliability of the approach taken by Johnson and VanDerWal [38] for several reasons:

1. First, their input data were binary, which is entirely legitimate, but nonetheless very easily demonstrated to have less descriptive ability, less sensitivity for detecting PTI changes, and result in a greater opportunity for erroneous inferences [39,40].

2. Second, inappropriately pooling or comparing indices across seasons and habitats is widely condemned in methods texts because doing so disregards the known confounding effects of these variables on index values interpreted as relative abundance estimates (e.g. [30,41-43]). Doing so also ignores the large and demonstrable differences in mammal assemblages between habitat types reported in the two original studies Johnson and VanDerWal [38] reanalysed.

3. Third, transforming data points for a visual effect by dividing each PTI value by the highest PTI value injects dependency amongst ‘independent’ data, where the apparent shape of the relationship becomes dependant on the maximum value observed. It also unnecessarily adds another random variable to the list of potential confounding factors.

4. Fourth, true relationships between predators may not be linear, but performing analyses on only the few extreme PTI values wastefully disregards the remaining biologically meaningful data.

5. Fifth, chasing *p* values in such exploratory analyses is almost guaranteed to generate statistically significant results (where *p* = ≤0.05), yet these non-confirmatory results are known to often be biologically spurious [44].

6. Sixth, interpretation of such correlative data relies on *a priori* allocation of response (e.g. fox PTI) and predictor (e.g. dingo PTI) variables, but when these variables are reversed (a entirely plausible and legitimate approach), the correlative evidence is just as strong but the interpretation is the opposite; that is, foxes suppress dingoes [15].

Given these issues, reducing our experimental data to approach the subject matter in the correlative way described by Johnson and VanDerWal [38] would be inferior to the primary analytical approaches we have taken, which do permit conclusive and demonstrable statements about cause (i.e. dingo control) and effect (i.e. mesopredator release; [35]).

## Discussion

### Evidence for lethal control-induced mesopredator release

Our results provide demonstrable and conclusive evidence that in almost all cases and no matter which analytical approach was used to evaluate these experimental data, neither foxes, cats or goannas responded positively to contemporary dingo control practices. The three sympatric mesopredators we assessed were typically in similar or greater abundance in unbaited areas relative to baited areas (Tables 1 and 2). No short-term increases in mesopredator PTI values were observed in baited areas (Tables 3 and 4, Figure 3). Longer-term mesopredator PTI trends typically were either uncorrelated or positively correlated with dingo PTI trends over time (Table 5, Figure 2). If either fox, cat or goanna populations gained an overall benefit from lethal dingo control, then (1) overall mean mesopredator PTI should have been higher in baited areas, and/or (2) mesopredator PTI values should have increased in baited areas following baiting, and/or (3) mesopredator PTI trends should have diverged from dingo PTI trends over time. Rarely did any of these occur for any mesopredator at any site.

As found in wolves (e.g. [14,45]), it is possible that dingo populations subjected to lethal control may also undergo demographic and social changes that might benefit mesopredator populations, such as the loss of experienced adults and an associated reduction in dingoes’ ability to repel mesopredators [16,24]. However, dingo populations subjected to baiting are likely to contain a larger proportion of adults and adult-sized animals, exhibit temporarily elevated activity levels and territorial behaviour, and prey more heavily on species smaller than themselves [46]. Each of these behavioural changes would be highly unlikely to create a favourable environment for mesopredators attempting to establish in dingo-baited areas, where only individual dingoes from small and newly-formed packs are needed to kill invading foxes or feral cats [47]. Assessment of demographic perturbations to dingo populations was outside the scope of the present study, but if demographic and social changes to dingo populations occurred, they did not appear to benefit foxes, cats or goannas.

**Figure 6:**
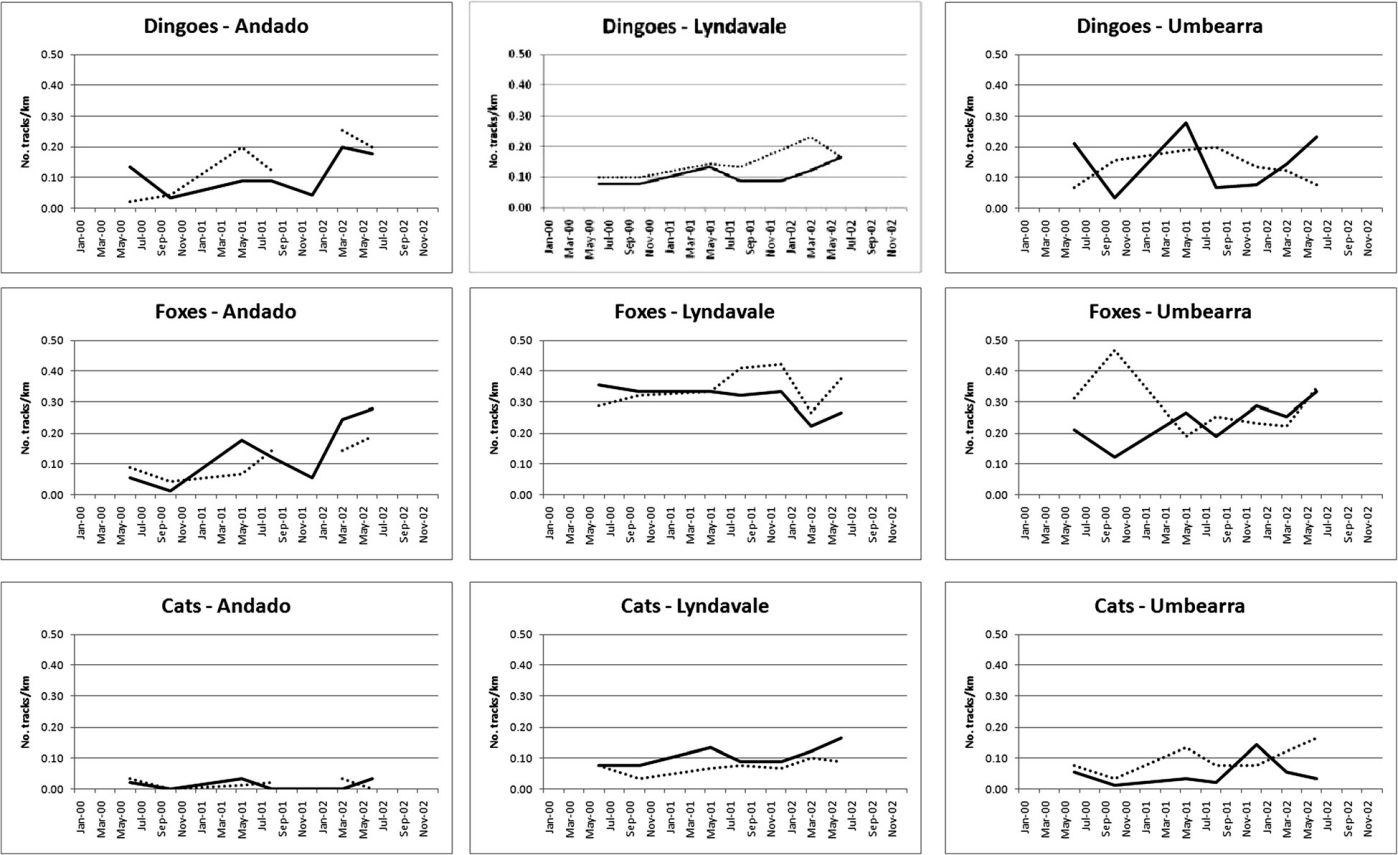
**Abundance trends of dingoes, foxes and cats in paired baited (solid lines) and unbaited (dotted lines) treatment areas at three sites in central Australia (adapted from **[48]**), showing no evidence of mesopredator release following dingo control.**

The study of Eldridge et al. [48] is the only other completed and true predator manipulation experiment investigating *in situ* dingo-mesopredator relationships [18,30]. Using a different PTI methodology, Eldridge et al. [48] likewise showed that fox and cat population trends did not diverge from dingo trends over time (Figure 6), nor were dingo and fox PTI values negatively correlated (*r* = 0.049, *p* = 0.588). Viewed together or separately, both these and our large-scale, multi-year, predator-manipulation experiments at 12 sites across the beef-cattle rangelands of continental Australia provide consistent and demonstrable results that do not support, and indeed contradict, perceptions (reviewed in [28,29]) that: (1) contemporary dingo control practices facilitate immediate or subsequent increases in mesopredator abundances, that (2) ceasing dingo control leads to reduced mesopredator abundances, or that (3) mesopredator populations are negatively associated with dingo populations over time. Long-term (10–28 years) correlative studies of dingoes support these experimental results (e.g. [49,50]). A large and growing body of evidence from other countries similarly report neutral or positive relationships between terrestrial top-predators and mesopredators (e.g. [7,51-55]). Thus, not only is there a clear absence of reliable evidence for dingo control-induced mesopredator release, but there is a strong and growing body of demonstrable evidence of absence for the same.

Our findings are in accord with what is known from other predator manipulation experiments worldwide: fauna at lower trophic levels are unlikely to respond positively to lethal control where multiple predators are removed (i.e. dingoes and foxes in our case), where the efficacy of predator removal is low (i.e. where predator populations quickly recover), and where the fauna are not the primary prey species of the predator [18]. That fox populations did not respond positively to poison-baiting is intuitive given that they are widely known to consume baits intended for dingoes and have similar rates of increase to dingoes (e.g. [19,56,57]). ‘Dingo baiting programs’ should therefore be better thought of as ‘dingo and/or fox baiting programs’ or ‘canid baiting programs’. It is theoretically possible that mesopredators may have increased in response to the removal of dingoes but that this response was masked by the fact that mesopredators may also have been simultaneously removed by baiting. Regardless, our data demonstrate that the *overall* population-level responses of mesopredators to the distribution of non-selective poison-baits were not positive. Though not susceptible to the toxin at the doses used in canid baits, goannas also consume baits. That foxes (e.g. [58]) and goannas (e.g. [59]) can reduce the number of baits available to dingoes may be one reason why dingoes were not demonstrably reduced at all sites. Our results for cats might also have been expected given that dingoes and cats are known to have mixed (usually neutral) relationships, which are typically weaker in places where foxes are present [29]. Where studied, neutral relationships between dingoes and goannas are also typical and independent of baiting [50].

### Mesopredator release theory and reality

Our findings do not contradict mesopredator release theory, but merely indicate that contemporary dingo control practices do not produce the conditions required to generate a mesopredator release effect. In other words, and apart from targeting foxes as well, periodic poison-baiting across areas up to 4,000 km^2^ does not appear to suppress dingo populations to levels low enough and long enough for mesopredators to exploit the situation (Tables 1, 2 and 3, Figures 2 and 3). That contemporary dingo control practices do not trigger a trophic cascade is likely due to rapid reinvasion of dingoes back into baited areas, which typically occurs within weeks or months after baiting ([19]; present study). In the beef-cattle rangeland systems we studied, dingoes from higher-density populations are known to migrate >550 km in 31 days or >1,300 km in four months into areas with lower dingo densities, with approximately 15% of dingoes dispersing over 100 km [60]. However, such long-distance migrations are not usually required for dingoes to recolonise baited areas because baiting programs seldom reduce extant dingo populations by even 50% and source populations of dingoes may only be a few kilometres away, and even within the baited area ([22,61,62]; present study). This is dissimilar to historical dingo control practices (which were reliant on extensive exclusion fencing to inhibit recolonisation) which did enable extermination of dingoes from some areas [20,21]. The differences in overall efficacy (at reducing dingo abundance) between historical and contemporary control strategies could have contributed to the coarse continental-scale pattern of inverse relationships between dingoes and foxes often touted (e.g. [33,63]).

Contemporary canid control practices at our study sites, which represent contemporary land use across much of the Australian continent, may be defined as the spatiotemporally sporadic application of relatively minor amounts of poisoned bait throughout a mosaic of baited and unbaited areas [22]. Though we cannot tell, we expect the results we describe to occur commonly in places where contemporary canid control is practiced in this way. Although canid control temporarily removes some dingoes (and might change their social structures), as intended, this does not imply their complete or sustained eradication, which might produce a positive response from mesopredators in places it could actually be achieved. Thus, there might be a baiting-induced low-point where top-predator populations become ecologically ineffective in their roles [17,24]. However, our data suggest that this low-point may have to be extremely low given that dingoes were suppressed by up to 77% (Table 1) without corresponding evidence of positive responses from any mesopredator (Tables 3 and 5, Figures 2 and 3). Allowing dingo populations to recolonise following periodic suppression may provide livestock producers with a window of opportunity to reduce livestock depredation during high-risk times (such as peak cattle calving season) while retaining the ecological functions of dingoes over longer timeframes.

### Factors affecting predator responses to lethal control

Although our experiments were conducted over similar timeframes to most other predator manipulation experiments [18], it might be argued that 2–5 years is not long enough to detect positive mesopredator responses to dingo control. However, three lines of evidence suggest this is not the case for our data. First, the PTI methodology we applied was sufficient to detect the responses of predators to the bottom-up effects of rainfall within the timeframe covered ([22,46,64]). Some also claim that the top-down effects of dingo control can be greater than the bottom-up effects of rainfall in the systems we studied [65], so the predicted positive responses of mesopredators to baiting should have been observable. Second, from small-scale observational studies conducted in similar habitats to ours, Pettigrew [27], Christensen and Burrows [25] and Lundie-Jenkins et al. [26] each reported detectable positive responses of foxes and/or cats within a few weeks or months after single dingo-control events, implying that 2–5 years of repeated baiting and population monitoring across spatial scales several orders of magnitude larger should have readily detected both acute and chronic mesopredator releases. Third, having been exposed to the same treatments for at least 10 years at the three Blackall sites (where predator abundances might be expected to have stabilised), mesopredator abundances should have been higher in baited areas, but they were not (Tables 1 and 2). In contrast, the only two (out of a possible 26) instances where a sympatric mesopredator was detected more frequently in baited areas at any site was for goannas at Blackall and Tambo – sites where dingo abundances were not demonstrably less in baited areas (Table 1). These lines of evidence indicate that our methodology was sufficient to detect immediate and longer-term increases in baiting-induced mesopredator activity or abundance if they were occurring.

Although we undertook our study in an experimental framework inclusive of buffer zones to maintain treatment independence, it is also important to remember that our approach was an evaluation of the overall population-level responses of mesopredators to contemporary top-predator control practices under real-world environmental conditions where dingoes, foxes, cats and goannas are each capable of dispersal and migration between treatments over time. In other words, we sought not to compare nil-treatment areas to paired treated areas with ‘X% reduction of dingoes’ or ‘X density of baits’, but with ‘contemporary dingo control practices’. This applied-science focus therefore produces results that reflect the *in situ* outcomes of contemporary dingo control practices in the beef-cattle rangelands present across much of the Australian continent. Alternative dingo control strategies which actually achieve complete and sustained dingo removal from the landscape (such as exclusion fencing) may yield different results, as may studies interested in smaller spatial scales where physical interactions between predators might be observed.

Viewed collectively, possible explanations for our observations might include that dingoes do not interact strongly with foxes, cats or goannas and/or that abundances of these mesopredators are associated primarily with bottom-up factors (such as rainfall, primary productivity, habitat complexity or prey availability), as has been found in other fox and cat studies (e.g. [66,67]). These bottom-up factors likely affect dingoes similarly [20,68]. Mesopredators (especially scavenging foxes and goannas) may also derive substantial benefit from dingoes through kleptoparisitism [19,69], which may also have contributed to our observations. The relative strength of top-down and bottom-up processes affecting predator populations in Australia has not been well studied, though it seems clear from our results that mesopredator populations in the rangelands do not appear to be enhanced by contemporary dingo/fox control practices. Indeed, we found no empirical evidence to support the supposition that cessation of periodic top-predator control will somehow suppress sympatric mesopredators, nor did we find evidence to suggest that commencement of top-predator control increases mesopredator activity or abundance. It is clear that a far greater understanding of context-dependant top-down and bottom-up processes must be acquired before biodiversity restoration is to be achieved simply by bolstering top-predator populations in Australia [19]. Thus, proposals to cease dingo control are presently unjustifiable on biodiversity protection grounds [70].

## Conclusions and implications

Our results provide strong, experimental evidence that contemporary dingo control practices do not produce immediate or sustained positive overall responses from foxes, cats or goannas in the beef-cattle rangelands of Australia, nor do they show that cessation of dingo control reduces mesopredator abundances. These findings increase our understanding of the potential indirect effects of periodic top-predator suppression on prey fauna at lower trophic levels and have important implications for dingo and threatened fauna management strategies. Some have asserted that simply ceasing lethal dingo control (aimed at protecting livestock) is a cost-effective strategy able to increase the abundances of threatened prey fauna populations of concern (e.g. [16,32,33,65]), but our results suggest that this is a utopian idea unlikely to produce such outcomes. Moreover, increasing the number of generalist predators – by encouraging dingoes in places with extent foxes and cats – typically widens the suite of prey vulnerable to unacceptable levels of predation [71-73]. Additional experimental studies on the indirect effects of dingo control practices on prey fauna (R6 in Figure 1) would be needed to verify or refute this prediction [15]. Our experimental results compel us to assert, as have others (e.g. [19,67,70]), that proposals to cease dingo control are presently unjustified on grounds that contemporary dingo control somehow releases mesopredators and threatens prey fauna through trophic cascade effects. In contrast, cessation of dingo control may actually benefit mesopredators that are also targeted by baiting and/or derive benefit from dingoes. Contemporary top-predator control might continue to be practiced for protection of livestock and native fauna in ways not incompatible with biodiversity conservation.

## Materials and methods

### Study sites and design

**Figure 7:**
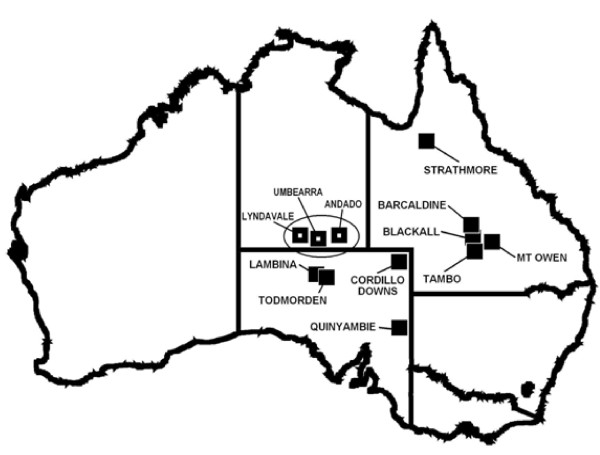
**Location of the nine study sites used in this study, and the three sites used in the study of Eldridge et al. (**[48]**; circled).** Each site studied was 100–1,500 km apart, except in the case of Todmorden and Lambina, which were neighbouring properties.

We conducted a series of large-scale, multi-year, predator-manipulation experiments (from [22,46,64]) on extensive beef-cattle producing properties in five different land systems representing the breadth of the beef-cattle rangelands of Australia, where mean rainfall varied from 160–772 mm annually, or from arid to tropical areas (Figure 7, Table 6).

**Table 6:** Study site and sample size details

**Site**	**Surveys**	**Period**	**Location**	**Land system**	**Combined size of treatment and nil-treatment areas**	**Dingo control history (previous 10 yrs)**	**Experimental design (rank of inference)**	**Mean annual rainfall (mm)**	**Plot-nights**		**Number of predator tracks observed**							
											**Baited areas**				**Unbaited areas**			
									**Baited**	**Unbaited**	**Dingo**	**Fox**	**Cat**	**Goanna**	**Dingo**	**Fox**	**Cat**	**Goanna**
Barcaldine	23	8/03 to 7/06	23.38′S, 145.37′E	Dry wood/grassland	5000 km^2^	Treatments intact for over 10 years	Quasi-experiment type l (5)	503	3015	3131	110	5	79	22	115	5	94	48
Blackall	23	8/03 to 7/06	24.23′S, 145.37′E	Dry woodland	5000 km^2^	Treatments intact for over 10 years	Quasi-experiment type l (5)	536	809	3271	28	1	18	18	145	18	52	16
Cordillo	7	7/08 to 11/10	26.21′S, 140.48′E	Sandy/stony desert	5300 km^2^	Both treatments previously exposed to opportunistic shooting only	Unreplicated experiment (3)	167	900	900	113	1	1	3	317	84	3	11
Lambina	6	6/09 to 12/10	26.54′S, 134.30′E	Sandy/stony desert	3800 km^2^	Opportunistic shooting and periodic baiting in both treatments	Classical experiment (1)	180	750	750	21	10	10	5	89	5	8	12
Mt Owen	19	5/94 to 7/98	25.51′S, 147.36′E	Dry woodland	800 km^2^	Opportunistic shooting in both treatments, no baiting in previous three years	Unreplicated experiment (3)	575	4389	4350	1240	16	425	217	2884	9	421	207
Quinyambie	14	4/08 to 8/11	30.33′S, 140.42′E	Sandy desert	4500 km^2^	Both treatments previously exposed to opportunistic shooting only	Unreplicated experiment (3)	160	1400	1400	447	47	3	2	297	103	8	0
Strathmore	9	7/95 to 9/98	17.37′S, 142.40′E	Tropical savannah	9000 km^2^	Opportunistic shooting and periodic baiting in both treatments	Unreplicated experiment (3)	772	2066	2186	291	0	250	110	509	0	291	152
Tambo	16	3/04 to 8/06	24.51′S, 146.36′E	Dry woodland	5000 km^2^	Treatments intact for over 10 years	Quasi-experiment type l (5)	532	1352	2130	357	0	63	52	464	3	123	23
Todmorden	11	8/08 to 11/10	27.80′S, 134.45′E	Sandy/stony desert	7200 km^2^	Opportunistic shooting and periodic baiting in both treatments	Classical experiment (1)	180	1300	1300	51	7	17	18	254	20	7	15
**Total**	**128**	**31**	**9**	**5**	**45,600 km**^**2**^			**160-772**	**15981**	**19418**	**2607**	**80**	**849**	**429**	**5450**	**222**	**1000**	**469**

Descriptions of experimental designs and rank of inference (1 = highest, 16 = lowest) are found in [35].

**Figure 8:**
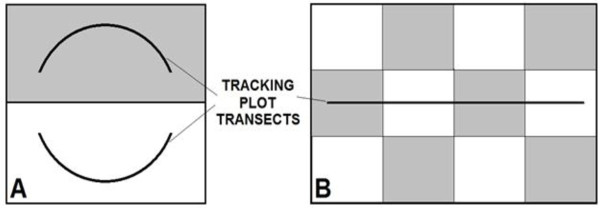
**Schematic representation of the experimental designs used at (A) Mt Owen, Strathmore, Quinyambie, Cordillo Downs, Todmorden, Lambina and (B) Barcaldine, Blackall and Tambo, showing the dispersion of baited (grey) and unbaited (clear) areas.** Baited and unbaited areas in design A were located on the same property. Baited and unbaited areas in design B represent adjacent properties.

Using paired nil-treatment areas without dingo control for comparison (Figure 8A), we examined the relative abundances of predators in paired areas subjected to periodic broad-scale poison-baiting for dingoes at six of nine study sites (Strathmore, Mt Owen, Cordillo Downs, Quinyambie, Todmorden, and Lambina; Table 6), referred to as the six experimental sites. Aerial and/or ground-laid sodium fluoroacetate (or ‘1080’) poison-baits were distributed individually (spaced at least 300 m apart) along landscape features (e.g. drainage lines, ridges, fragment edges etc.) and/or unformed roads according to local practices and regulations up to five times each year (typically once in spring and again in autumn at the six experimental sites, and every 2–4 months continuously at the other three sites). Baits were distributed over a 1–2 day period to a midway point in the buffer zone between treatments (described below; Figure 8A). Each bait weighed 100–250 g and contained at least 6 mg of 1080, sufficient to kill adult dingoes, foxes or cats (but not goannas) if consumed soon after bait distribution [57]. Such baiting practices are common, occur widely across Australia, and are considered the only effective dingo and fox control tool used in rangeland areas [56]. Cat and goanna populations are not typically susceptible to such baiting practices because goannas are tolerant of the toxin (at the low-level doses used in canid baits) and cats rarely consume carrion-like baits, preferring live prey instead (e.g. [59,74-76]). Opportunistic shooting of dingoes occurred at some sites during the study and historically (Table 6), but with negligible effects on dingo populations because very few dingoes were ever shot [22]. Experimental treatment (i.e. baited) and nil-treatment (i.e. unbaited) areas were randomly allocated. Treatment and nil-treatment areas were also replicated in some land systems (Table 6). Hone [35] defines this study design as an ‘unreplicated experiment’ or a ‘classical experiment’ for our site with replication (i.e. Todmorden and Lambina might be considered a single site with two treatments and two controls). Both treatment and nil-treatment areas at some of the six experimental sites were historically exposed to baiting up until the commencement of the experiment, whereas, both treatment and nil-treatment areas were not historically exposed to baiting at other sites (Table 6). Such histories were necessary to investigate the responses of predators to either the commencement or cessation of baiting, or to the ‘removal’ or ‘addition’ of predators (i.e. dingoes and foxes were killed at some sites or allowed to increase at others).

All procedures described were sanctioned by the relevant animal care and welfare authorities for each site (Queensland Department of Natural Resources’ Pest Animal Ethics Committee, PAEC 930401 and PAEC 030604; South Australian Department of Environment and Heritage’s Wildlife Ethics Committee, WEC 16/2008).

The other three sites (Barcaldine, Blackall and Tambo; referred to as the Blackall sites) were monitored for a similar length of time (Table 6), but differed from the six experimental sites in that the treatments and nil-treatments had already been established for over 10 years and they did not have buffer zones between them (Figure 8B). This allowed an assessment of the longer-term outcomes of lethal dingo control. Treatment size, independence and baiting practices therefore varied between the nine sites in order to deliver *in situ* tests which reflected contemporary dingo control practices within each bioregion. Experiments were conducted at large spatial scales, where the size of the total treatment and nil-treatment area at each of the nine sites ranged between 800 km^2^ and 9,000 km^2^, or 45,600 km^2^ in total (Table 6). The largest contiguous baited area was ~4,000 km^2^. By comparison, the combined size of the areas we assessed is approximately four times the size of Yellowstone National Park or half the size of the Greater Yellowstone Ecosystem, where a substantial amount of similar research on wolves has been conducted. Only one other predator manipulation experiment (on wolves and ungulates in Canada) has a larger spatial scale than ours (i.e. [12], a single site with one treatment area and three controls totaling ~60,000 km^2^), and very few such experiments ‘add’ predators; most ‘remove’ them [18]. Each site we studied was separated by 100–1,500 km, except in the case of Todmorden and Lambina, which were neighbouring properties (Figure 7).

### Predator population monitoring

Dingo and sympatric predator populations were simultaneously monitored in treatment and nil-treatment areas using passive tracking indices (PTI; [77]). This technique is the recommended and standard monitoring technique used for assessing terrestrial predator populations in Australia [78] and has also been used to monitor a variety of other predators in other countries (e.g. [79-81]). Variants of this technique are commonly used to sample many different terrestrial fauna around the world [82].

PTI surveys were conducted several times each year at each site and were repeated at similar times each subsequent year over a 2–5 year period (Table 6). At the Blackall sites, between 92 and 166 passive tracking plots (or ‘sand plots’) were spaced at 1 km intervals along unformed vehicle tracks. At the six experimental sites, 50 plots each were similarly established in both the treatment and nil-treatment areas (i.e. 100 plots per site). The number of plots we monitored is roughly double that used in most other similar studies of dingoes and we also monitored these plots for longer than most similar studies at most of our sites [30]. For any given survey, plots in both treatments were read and refreshed at the same time daily by the same experienced observer and were monitored for up to 10 successive days (usually 2–5). The location of the first tracking plot in each treatment area was randomly allocated and plots were distributed throughout a similar suite of microhabitat types in both treatment areas to minimise potential microhabitat differences in predator detectability between treatments. Plots rendered unreadable by wind, rain or other factors were excluded from analyses. All predator track intrusions were counted (i.e. a continuous measure). PTI values for a given survey therefore represented the mean number of predator track intrusions per sand plot tracking station per 24 hr period (i.e. the mean of daily means; [77]). Analysed appropriately, PTIs collected in this way can be interpreted as robust estimates of relative abundance (e.g. [40,41,83]).

At least one PTI survey was conducted before the imposition of treatments (i.e. before commencement or cessation of baiting in a given treatment) at the six experimental sites to identify any spatial variation in predator population abundances between treatments prior to manipulations. Tracking plot transects at these six sites were separated by a buffer zone 10–50 km wide (Figure 8) to achieve treatment independence during individual surveys. The appropriate width of the buffer zone at each site was based on the width of 1–2 dingo home ranges in the study areas (e.g. [61,62]). Tracking plots were located no closer than 5–25 km from the edge of the treatment area (i.e. half the width of the buffer zone) to minimise potential edge effects. Overall, we obtained 35,399 plot-nights of tracking data from 128 surveys conducted over 31 site-years (Table 6).

### Analytical approaches

We used three primary approaches to examine the effects of lethal dingo control on sympatric mesopredators. First, we compared the mean PTI of predators (both overall, and also stratified by season) between baited and unbaited areas at each site using repeated measures ANOVAs. Second, we determined the short-term changes in predator PTI values between pre- and post-baiting surveys (conducted within four months since dingo control) through a (1) 2-factor (time and treatment) repeated measures ANOVA, and for completeness, by also (2) assessing mean net changes in PTI (i.e. changes in the baited area after accounting for changes in the unbaited area) with two-tailed t-tests for each site where at least four pre- and post-baiting surveys were conducted. Third, we assessed temporal correlations between abundance trends of dingoes and each sympatric mesopredator, separately for baited and unbaited areas. Data were not available for goannas in winter or foxes at one site in northern Australia, because ectothermic reptiles are typically inactive in winter and the national distribution of foxes did not extend to the northernmost site (Strathmore) in the Gulf of Carpentaria (Figure 7). Additional details on study site characteristics, dingo control history, experimental designs, bait types and densities, baiting regimes and efficacy, and application of passive tracking indices can be found in Table 6 or in Allen [46,64] and Allen [22].

Extant mesopredators have been predicted to respond positively to dingo control through either (1) a numerical reduction in dingo abundance and/or, where sustained numerical reductions may not have occurred, through (2) behavioural or demographic changes to dingo populations which facilitate increases in mesopredator activity or abundance (e.g. [16,24,65]). These responses are predicted to occur rapidly, manifest first by immediate and then sustained increases in mesopredator PTI (e.g. [25-27]). Thus, our primary analytical approaches would detect dingo control-induced mesopredator release where: (1) mean mesopredator PTI is greater in paired baited areas (indicative of greater mesopredator densities in baited areas); where (2) mesopredator PTI increases in baited areas (relative to unbaited areas) shortly after dingo control (indicative of an immediate behavioural release of mesopredators); or where (3) mesopredator PTI trends diverge over time (indicative of a longer-term numerical release of mesopredators). An alternative approach to assessing dingo-mesopredator relationships has been suggested by Johnson and VanDerWal [38], which report the occurrence of non-linear ‘triangular relationships’ between dingoes and foxes; dingoes apparently setting an upper limit on fox abundance in temperate forests. This approach pools binary data across sites and surveys, transforms the values to generate a visual affect, selects only the extreme values, and then correlates these extreme values between predators. For completeness, we therefore explored the utility of this approach with our data as a supplementary exercise only.

## Competing interests

The authors declare that they have no competing interests.

## Authors’ contributions

LA designed and supervised the study. BA and LA collected field data and performed preliminary analyses. BA performed the remaining analyses, constructed the tables and figures and wrote the majority of the manuscript. RE performed statistical analyses. LA, RE and LL contributed further to the writing of the manuscript. No conflict of interest is declared. All authors read and approved the final manuscript.
